# Understanding the drive to escort: a cross-sectional analysis examining parental attitudes towards children’s school travel and independent mobility

**DOI:** 10.1186/1471-2458-12-862

**Published:** 2012-10-11

**Authors:** George Mammen, Guy Faulkner, Ron Buliung, Jennifer Lay

**Affiliations:** 1 Faculty of Kinesiology and Physical Education, University of Toronto, 55 Harbord St, Toronto, ON, M5S 2W6, Canada; 2 Department of Geography and Program in Planning, University of Toronto, 100 St.George St, Toronto, ON, M5S 2W6, Canada; 3 Metrolinx, 20 Bay St. Suite 600, Toronto, ON, M5J 2W3, Canada

**Keywords:** Independent mobility, Active school transportation, Unescorted/escorted children, Physical activity, School travel, Walking school bus

## Abstract

**Background:**

The declining prevalence of Active School Transportation (AST) has been accompanied by a decrease in independent mobility internationally. The objective of this study was to compare family demographics and AST related perceptions of parents who let their children walk unescorted to/from school to those parents who escort (walk and drive) their children to/from school. By comparing these groups, insight was gained into how we may encourage greater AST and independent mobility in youth living in the Greater Toronto and Hamilton Area, Canada.

**Methods:**

This study involved a cross-sectional design, using data from a self-reported questionnaire (*n* =1,016) that examined parental perceptions and attitudes regarding AST. A multinomial logistic regression analysis was used to explore the differences between households where children travelled independently to school or were escorted.

**Results:**

Findings revealed that unescorted children were: significantly older, the families spoke predominantly English at home, more likely to live within one kilometer from school, and their parents agreed to a greater extent that they chose to reside in the current neighborhood in order for their child to walk to/from school. The parents of the escorted children worried significantly more about strangers and bullies approaching their child as well as the traffic volume around school.

**Conclusions:**

From both a policy and research perspective, this study highlights the value of distinguishing between mode (i.e., walking or driving) and travel independence. For policy, our findings highlight the need for planning decisions about the siting of elementary schools to include considerations of the impact of catchment size on how children get to/from school. Given the importance of age, distance, and safety issues as significant correlates of independent mobility, research and practice should focus on the development and sustainability of non-infrastructure programs that alleviate parental safety concerns.

## Background

Physical inactivity in children and youth continues to be an international epidemic, with the potential of contributing to chronic disease in adulthood. For example, only 9% of Canadian boys and 4% of girls are accumulating the recommended amount of physical activity (PA) (i.e. 60 minutes of moderate-vigorous physical activity per day) needed for improved health [1]. Given that the majority of children and youth are not meeting PA guidelines, much attention has been placed on strategies to elevate PA in this population. Promoting Active School Transportation (i.e., walking/biking to and from school; AST) may be one strategy for increasing youth PA levels.

Research has demonstrated many associated physical benefits of AST including achieving greater volumes of overall PA, healthier body compositions, and higher levels of cardiorespiratory fitness [2-6]. Beyond these potential physiological benefits, more recent research indicates a significant relationship between AST and cognitive performance. Students travelling actively to school have been shown to perform better academically, and appear to experience less stress (perceived) when confronted with cognitive challenges during the school day [7-9]. The sort of environmental engagement that AST (walking in particular) facilitates has also been shown to improve levels of attention, memory, and energy, and reduce levels of anxiety, fatigue, anger, and sadness [10]. For example, Fusco et al. [11] reported that positive affect might be triggered by children’s increased cognizance of, and experience with visually stimulating aspects of the environment such as gardens, trees, and flowers.

Despite these benefits, evidence suggests that there has been a decline in AST among children internationally. For example, in the US, 12.7% of K–8 (i.e., 6–14 years) students usually walked or biked to school in 2009 compared with 47.7% in 1969 [12]. This decline is also evident in some low- and middle-income countries. In Vietnam, there was a decrease in the prevalence of AST in adolescents from 27.8% in 2004 to 19.6% in 2009 [13]. Within the Greater Toronto Area, Canada’s largest metropolitan region, there was an approximate 10% decline between 1986 and 2006 (53% to 42% for 11–13 year olds, 39% to 31% for 14–15 year olds) [14].

Concern with low levels of PA and declining AST underlies research into the potential barriers that could explain why more children are being driven to school today than in the past. The increase in car use has been noted to stem from issues of the built environment, such as poor road and sidewalk infrastructure [15] and negative parental perceptions regarding AST safety [16,17]. However, a more consistent barrier pertains to a school’s distance from home [18-20]. For example, one U.S report [21] explained that nearly 35% of children lived within a one-mile radius from their school in the late 1960s. By the year 2000 this proportion had declined to 20%, and this decline is one primary contributing factor to lower levels of AST. The median distance between home and school for US children aged up to 15 years has been described as two miles (i.e., 3.2 km) [22]. Wong, Faulkner, and Buliung [20] however, uniquely highlight the importance of considering the complexity of the ‘distance’ phenomena. Distance between home and school is ‘produced by interactions between complex social and economic processes that influence home and school locations’ ([20] p.15.).

Despite the fact that parents consistently cite distance as the number one barrier to their children actively commuting to school, many children actually live within a reasonable walking distance from school, which has been generally established to be within a 2 km radius [23]. For instance, a British study found that 50% of children being driven to school lived within one mile [24]. An Australian study showed that 80% of students lived within one kilometer from school, yet 47% of these students were driven [25]. As noted by Fotel & Thomsen [26] findings like this indicate that the increasing prevalence of car use is resulting in less AST in children, and additionally a decrease in children’s independent mobility. Targeting families who live within reasonable walking distances from their respective schools, for AST interventions, could simultaneously help elevate overall levels of AST, and foster greater independence in children.

Independent mobility, another associated benefit of AST, in children and youth has been defined as actively traveling to a destination (e.g., to and from school) without adult supervision [27]. Being unescorted generates greater opportunities for children to be active, particularly since they are not dependent on adults to leave the vicinity of their homes. Subsequently, independent mobility has been found to foster personal growth and development, by helping children develop road and traffic safety skills, motor skills [28], and higher acquisition, processing and structuring of environmental knowledge [29]. In addition, independent mobility also helps children socialize with their peers and helps develop emotional bonds between children and the natural environment [28]. Working against independent mobility, is parental, caregiver, and even community anxiety (perhaps intensified through media exposure) with respect to the safety of children and youth [30].

Although the personal benefits of children’s independent mobility can be partially accrued through AST, international data shows a decline in not solely AST, but independent mobility as well [31]. Research suggests the decline in AST may be more of a reflection of the decrease in independent mobility and this decrease may be associated with parental AST safety concerns [16,17]. This speculation corresponds with one AST based framework [32], which postulates that parents make the ultimate decision regarding their children walking/biking to/from school. This decision, however, is influenced by their perceptions of the physical and social environmental factors, and parents are more likely to allow their children to practice AST if they themselves have positive attitudes and perceptions pertaining to active transport.

However, the broader AST literature typically associates correlates with the school travel mode choice only and does not consider that the escort decision and travel mode decision may be underpinned by different parental decision making processes [33]. Research in this area has examined demographic characteristics of families and children who are escorted/unescorted to school [34,35], but has failed to focus on distinguishing parental AST related attitudes/perceptions of those children who are unescorted (typically by walking) to school from those who are escorted to school by any means. In addition, Sharpe and Tranter [36] argue that the literature has typically conflated AST and independent mobility as similar constructs. There is a need to distinguish between these constructs in addressing the potentially different challenges in encouraging both AST and independent mobility. Doing so may provide additional insight into how mode shift could be enabled within households living a walkable distance from school, with a view to simultaneously encouraging AST and independent mobility.

While independent mobility is generally operationalized as actively commuting without an escort, research has shown that being escorted while walking may also produce the associated benefits of independent mobility in children. For example, Sissons-Joshi and colleagues [37] discovered that children who walked escorted by an adult possessed greater environmental knowledge in relation to the children who travelled to school unescorted. Sissons-Joshi et al. commented that an adult presence en route to school can heighten children’s awareness regarding the surrounds as they engage in environment related discussions. Interventions to promote AST might then be tailored to shift school travel behavior from driving, to escorted walking, to independent walking of the child.

Thus, in an effort to explore independent mobility among children, the primary objective of this study was to examine differences in family characteristics and parental AST related attitudes/perceptions between unescorted walkers (i.e., independently mobile children), escorted walkers (i.e., children walking to school with an adult), and escorted drivers (i.e., children driven to school by their parents) living within two kilometers from school. A secondary objective was to examine perceptions of strategies that would increase the likelihood of AST among the escorted drivers.

## Methods

### Study design

This study involved a cross-sectional design, using data from a self-reported survey conducted by Metrolinx in the Greater Toronto and Hamilton Area (GTHA), Canada. Metrolinx is the regional transportation agency for the GTHA. With a population exceeding 6.2 million, the GTHA is Canada’s largest and most socially and culturally diverse urban region. The region contains Canada’s largest city, the City of Toronto, and some of Canada’s newest and largest (by population) suburban cities, such as Mississauga. Politically, the region contains several municipal governments (Toronto, Hamilton, Missisauga) and regional municipalities (e.g., Durham, Halton, Peel and York). There is an interesting juxtaposition of built forms to be found across the region. Many traditional neighborhoods with colonial origins traceable to the late 18^th^ and early 19^th^ centuries can be found within city (e.g., the older neighborhoods of Toronto and Hamilton) and suburb alike (e.g., Streetsville, Mississauga), alongside Canada’s first early experiments with a more modern type of suburban, car-centred, development. For example, the corporate new towns/suburbs of Don Mills in Toronto (1952–1965) and Erin Mills in Mississauga (built from the early 1970s to 1980s). Pockets of traditional form in the outer suburbs, however, are like monuments to the past, often surrounded by a lower density residential monoculture served by auto-oriented retail (e.g., strip malls, super regional malls, and big-box stores). The City of Toronto’s urban geography also includes a relatively dense inner suburb of former municipalities politically amalgamated with “old” Toronto in 1998, the most recent example of a political and geographical expansion of city limits traceable to its incorporation in 1834.

Notably, urban form, and street design in particular, across the region, matches, in many ways, the technological evolution of transport technologies and systems, and expectations of particular political administrations, over time, regarding what could or should be the dominant mode(s) of private and public transport. There are, then, older neighborhoods that predate mass automobile consumption, where walking, carriage, perhaps the omnibus, and later the horsecar (later still the electrified streetcar) were the only, if not preferred transport options at the time. These types of neighbourhoods contrast, in many ways, with those places planned and built during the post World War II era. That is not to say that some people in the region’s auto-oriented neighborhoods do not use other modes, they do – but in the presence of an urban fabric not adequately designed to support something other than the car [38].

The purpose of the questionnaire was to gain insight into school travel behaviour and parental perceptions and attitudes regarding AST in the GTHA. In order to standardize the administration of the survey, and to ensure an effective balance across each elementary school grade, the survey focused on the household’s eldest child attending elementary school between kindergarten to grade 8 (i.e., 6–14 years old). The sample was drawn from Harris/Decima’s ASDE Survey Sampler database based on Census Division (CD) from the 2006 Census. The final dataset was weighted by the child’s gender and grade, and by CD. A total of 1,016 interviews, with a response rate of 40.3%, were conducted in each of the following four areas: the City of Toronto (*n* =285), the City of Hamilton (*n*= 250), the Region of Peel (*n*=255) and the combined regions of Durham, Halton and York (*n*=226). Over half of this total sample (*n*= 564) lived within 2 km from school. Voxco CATI software was used in conducting computer-aided telephone interviews. Ethical approval for this secondary data analysis was granted by the institutional research ethics committee at the University of Toronto.

### Measures

#### Travel mode

Parents were asked to report how their child usually travels to/from school. Response items included: Driven- by a member of the household or family member, driven- as part of a carpool with neighbors or friends, by school bus, by public transit-bus, by public transit-subway or streetcar, by walking, by cycling, and other. The prevalence of the various transportation modes among those living within 2 km from school were: walking (n=344; 61%), driving (n=146; 26%), school bus (n=73; 13%), public transit (n=6; 1%), biking (n=6; 1%), and other (n=10; 2%). Although we acknowledge that AST entails both walking and biking to/from school, only 1% of the sample living within 2 km from school used biking as a primary mode choice and thus were excluded for analysis. Parents were also asked if their children were accompanied by an adult en route to school. Thus to address our objectives of examining differences among unescorted walkers (*n*=111), escorted walkers (*n*=233), and escorted drivers (*n*=146), only respondents living within 2 km from school were selected for analysis (*n*=490). On the questionnaire, self-report distance to school was used, fixed to three intervals: a) < 1 km; b) 1 to 2 km and c) > 2 km. For our analysis, we excluded those who lived more than 2 km from school.

#### Demographics

Child, parental, and household demographics were collected. Child characteristics included: age, gender, and grade (Primary: kindergarten to grade 3 (i.e., ages 6–9); Junior Grades 4–6 (i.e., ages 10–12); Senior: Grades 7 and 8 (i.e., ages 13 and 14)). Parental characteristics included: age (year of birth), gender, employment status (full-time vs. other), and school travel mode as a child (walking or other). Household demographics included: household income (<$60K, >$60K), number of licensed drivers, number of cars owned, primary language spoken at home (English vs. other), distance from school (<1 km, 1-2 km), and place of residence, at the scale of the city or regional municipality (City of Toronto, City of Hamilton, Region of Peel, and the combined regions of Durham, Halton and York).

#### Attitudes/perceptions of walking to school

Parental attitudes regarding perceived AST safety, perceived AST advantages, and methods to increase greater AST were assessed between the three target groups. With regard to AST safety, parents were asked to respond to a 5-point Likert scale ranging from 1 (strongly disagree) to 5 (strongly agree) assessing constructs that related to: route safety, traffic safety, bullies/strangers, and discussions about AST safety with their child. Parents were also asked to report the age (child) at which they (the parent) would feel comfortable allowing their child to travel to school unsupervised.

With regard to perceived AST advantages, parents were asked to respond to a 5-point Likert scale ranging from 1 (strongly disagree) to 5 (strongly agree) assessing constructs that related to: the importance of exercise during the school trip and the importance of travelling to school in an environment-friendly way. Using the same scale, parents were also asked the extent to which the decision for living in their current neighborhood was based on whether their child could walk to/from school. An additional item pertaining to the convenience of their child’s current school travel mode was asked. The item was measured using a 4-point Likert scale (very inconvenient, somewhat inconvenient, somewhat convenient, very convenient) with high scores indicating greater convenience.

Concerning methods to increase greater AST, parents of the escorted drivers responded to two items asking how appealing it would be to walk with their child to school, and have them walk to school with a supervised group organized by the school. Response options for these two items ranged from 1 (Unappealing) to 4 (Appealing). Parents were then asked to respond to one item on a 5-point Likert scale ranging from 1 (strongly disagree) to 5 (strongly agree) to assess if a list of parents who would like to have their children walk together would be useful. Parents were also asked to respond to two items designed to assess the likelihood of utilizing services organized by the school that could potentially facilitate greater AST. These two items were assessed on a 5-point Likert scale ranging from 1 (not at all likely) to 5 (extremely likely). The final list of questions asked these parents to what extent would certain strategies increase the likelihood of their child actively travelling to/from school. These strategies included: a walking school bus organized by the school, before/after school supervision, a school closer to home, slower speed around school area, well-maintained sidewalks, school zone cautionary signs, police presence around school, and crossing guards/marked crossing by school. These items were assessed on a 5-point Likert scale ranging from 1 (would not increase likelihood at all) to 5 (would greatly increase the likelihood).

#### Statistical analysis

Descriptive statistics were used to examine demographic variables. The dependent variable in this study related to school travel mode (i.e., unescorted walkers, escorted walkers, and escorted drivers) among those living within two kilometers from school. Four groups of independent variables were explored in terms of demographics, measures of safety, measures of AST related advantages, and measures of methods to increase levels of AST. Since the dependent variable was a categorical variable with multiple levels, a multinomial logistic regression analysis was used to explore the differences between factors that effect school travel mode. Four separate logistic regression models were specified and estimated, each having the same dependent variable but specified using different groups of independent variables. A limitation of the sample size and missing values prevented model estimation using all variables simultaneously. Odds ratios and confidence intervals are reported; statistical analysis was conducted using IBM SPSS statistics 19 (IBM, PASW Statistic). An alpha level of .05 was used for all statistical tests.

## Results

### Descriptive overview

Of the total sample of 1,016 participants, 111 (10.9%) of parents living within 2 km of school had a child who walked to school unescorted, 233 (22.9%) had a child who walked to school escorted, and 146 (14.4%) had a child who was driven to school escorted. Table 1 displays demographic information pertaining to the child, parents, and household characteristics (note: frequencies and percentages are based on completed data). In terms of child characteristics, unescorted walkers were older and in higher grades. Unescorted walkers typically had older parents with full-time employment. Walkers (escorted or unescorted) typically lived closer to school when compared with children who were driven, and English was the predominant language spoken at home for unescorted walkers.

**Table 1:** Characteristics of unescorted walkers, escorted walkers, and escorted drivers

	***Unescorted walkers (n =111)***	***Escorted walkers*(*n*=233)**	**Escorted drivers (*n*=146)**
***Child Characteristics***			
Age (years), *M* ± *SD*	11.90 ± 1.60	7.64 ± 2.59	8.09 ± 2.62
Gender, *%* (*n)*			
Male	47.7% (52)	49.6% (115)	50% (73)
Female	52.3% (57)	50.4% (117)	50% (73)
Grade, *%* (*n)*			
Primary (Grades K-3)	2.7% (3)	69.4% (162)	66.1% (96)
Junior (Grades 4–6)	34% (37)	23.8% (55)	24.1% (35)
Senior (Grades 7–8)	63.3% (69)	6.8% (16)	9.6% (14)
***Parental Characteristics***			
Age (years), *M* ± *SD*	44.41 ± 7.21	40.51 ± 8.61	40.25 ± 5.51
Gender, *%* (*n)*			
Male	25.2% (28)	33.0% (77)	34.2% (50)
Female	74.8% (83)	67.0% (156)	65.8% (96)
Employment Status, *%* (*n)*			
Full-time	67.9% (74)	54.1% (120)	73.2% (104)
Other	32.1% (35)	45.9% (102)	26.8% (38)
School Travel Mode as a Child, *%* (*n)*			
Walking	79.3% (88)	80.9% (178)	65.7% (94)
Other	20.7% (23)	19.1% (42)	34.3% (49)
***Household Characteristics***			
Income, *%* (*n)*			
< $60K	36.6% (37)	39.1% (81)	30.1% (40)
> $60K	63.4% (64)	60.9% (126)	69.9% (93)
Cars: Licensed Drivers, *M* ± *SD*	1.79 +/−0.75	1.84 +/− 0.81	1.97 +/− 0.53
# of cars/household	1.44 +/− 0.74	1.28 +/− 0.75	1.70 +/− 0.53
Predominant Language Spoken			
English	79.1% (87)	59.7% (135)	57.9% (81)
At least One other	20.9% (23)	40.3% (91)	42.1% (59)
Distance from School			
< 1 km	72.7% (72)	71.4% (152)	36.3% (53)
1-2 km	27.3% (27)	28.6% (61)	63.7% (93)
Region			
Toronto	36.0% (40)	61.8% (144)	29.7% (43)
Hamilton	8.1% (9)	6.9% (16)	6.9% (10)
Peel	17.1% (19)	14.6% (34)	31.0% (45)
Durham, York, Halton	38.7% (43)	16.7% (39)	32.4% (47)

#### Group-wise comparisons on demographic characteristics

##### Escorted walkers vs. Unescorted walkers

Table 2 displays all multinomial analysis comparing demographic and parental attitudinal variables among the three target groups. Older children are significantly less likely to walk escorted than to walk unescorted, *B*=−0.92, *OR*= 0.40, Wald χ2 (1)=71.87, *p* <.001, and if the predominant language spoken at home is English, *B*=−1.23, *OR*= 0.29, Wald χ2 (1)=7.96, *p* <.01.

**Table 2:** **Self-reported attitudes and perceptions regarding active school transportation (Unescorted walkers,*n*=111, escorted walkers,*n*=233, escorted drivers,*n*=146)**

	***B*(SE)**	***Lower***	**95% CI For odds Ratio**	***Upper***
			***Odds Ratio***	
**Escorted Walkers vs. Unescorted Walkers**				
***Demographics***- Intercept	11.20 (1.67)***			
Child’s Age	-.92 (.11)***	.32	.40	.49
Gender (male)	.26 (.36)	.64	1.30	2.65
Parents Income (>$60K)	.60 (.47)	.71	1.8	4.63
Employment Status (full-time)	-.76 (.41)	.21	.47	.93
# of cars/household	-.57 (.32)	.30	.56	1.05
# of driver licenses	.41 (.30)	.84	1.51	2.71
Language Spoken in the Home (English)	-1.23 (.44)**	.12	.29	.68
Parental School Travel Mode as a Child (walking)	.85 (.46)	.96	2.35	5.75
School Distance from home (<1 km)	-.67 (.44)	.22	.51	1.21
Region (Toronto)	.28 (.71)	.33	1.32	5.34
(Peel)	-.06 (.79)	.20	.94	4.41
(Durham, York, Halton)	-1.00 (.74)	.09	.37	1.58
***Perceived AST Safety*** -Intercept	2.30 (1.13)*			
There are safe bike routes/paths around the school^1^	-.09 (.08)	.78	.92	1.07
People drive safely enough in my neighborhood^1^	-.18 (.09)	.70	.84	1.00
There are too many cars in the morning around my school^1^	.01 (.09)	.84	1.01	1.21
I worry about strangers/bullies approaching my child^1^	.26 (.09)**	1.08	1.30	1.56
I Have discussed how to walk/bike safely with child^1^	-.38 (.19)*	.47	.69	1.00
At what age did/would you allow your child to travel to school unsupervised?	.37 (.09)***	1.22	1.45	1.71
***Perceived AST advantages***- Intercept	1.66 (1.26)			
Importance of child exercising on route to/from school^1^	-.14 (.23)	.56	.87	1.35
Importance of child travelling to school in an environmentally friendly way^1^	.04 (.19)	.71	1.04	1.52
I chose to live in my area so my child could walk to school^1^	-.11 (.11)	.73	.90	1.12
Convenience of current method of school travel^2^	.01 (.09)	.84	1.01	1.21
***Escorted Drivers vs. Unescorted Walkers***				
***Demographic****s*- Intercept	10.88 (1.71)***			
Child’s Age	-.88 (.11)***	.34	.42	.52
Gender (male)	-.13 (.38)	.42	.88	1.86
Parents Income (>$60K)	.30 (.49)	.51	1.35	3.55
Employment Status (full-time)	-.05 (.45)	.40	.95	2.28
# of cars/household	.69 (.35)*	1.00	2.00	4.00
# of driver licenses	.09 (.32)	.59	1.10	2.05
Language Spoken in the Home (English)	-1.45 (.45)***	.10	.23	.56
Parental School Travel Mode as a Child (walking)	-.08 (.45)	.38	.93	2.25
School Distance from home (<1 km)	-2.24 (.45)***	.04	.11	.26
Region (Toronto)	-.56 (.75)	.13	.57	2.47
(Peel)	.26 (.80)	.27	1.30	6.15
(Durham, York, Halton)	-.65 (.76)	.12	.52	2.32
***Perceived AST Safety***- Intercept	2.14 (1.23)			
There are safe bike routes/paths around the school^1^	-.16 (.10)	.71	.86	1.03
People drive safely enough in my neighborhood^1^	-.00 (.11)	.81	1.00	1.24
There are too many cars in the morning around my school^1^	.28 (.12)*	1.04	1.31	1.67
I worry about strangers/bullies approaching my child^1^	.46 (.13)**	1.22	1.58	2.05
I Have discussed how to walk/bike safely with child^1^	-1.02 (.19)***	.25	.36	.52
At what age did/would you allow your child to travel to school unsupervised?	.39 (.11)***	1.20	1.48	1.83
***Perceived AST advantages***- Intercept	5.94 (1.33)***			
Importance of child exercising on route to/from school^1^	-.95 (.22)***	.25	.39	.60
Importance of child travelling to school in an environmentally friendly way^1^	.18 (.22)	.79	1.20	1.86
I chose to live in my area so my child could walk to school^1^	-.42 (.11)***	.53	.66	.82
Convenience of current method of school travel^2^	-.19 (.12)	.66	.82	1.04
***Perceived methods to increase greater AST***- Intercept	-3.01 (.87)***			
Appeal of walking to school with child^3^	.37 (.18)*	1.01	1.44	2.06
Appeal of child walking to school with a supervised group, organized by the school.^3^	.28 (.19)	.91	1.32	1.91
List of nearby parents who would like their kids to walk to school together would be useful^1^	-.078 (.15)	.69	.93	1.24
Likelihood of utilizing a service that provides you with matches to other parents in your area who may be able to walk with you and your child to/from school^4^	-.01 (.13)	.77	.99	1.27
Likelihood of utilizing an organized walking school bus^4^	.26 (.13)*	1.00	1.30	1.68

^1^Scored on a 5-point scale with the anchors: 1 (strongly disagree), 2(somewhat disagree), 3 (neither agree/disagree), 4 (somewhat agree), 5(strongly agree).

^2^Scored on a 4-point scale with the anchors: 1 (Very inconvenient), 2(somewhat inconvenient), 3(somewhat convenient), 4(very convenient).

^3^Scored on a 4-point scale with the anchors: 1 (unappealing), 2(somewhat unappealing), 3(somewhat appealing), 4(Appealing).

^4^Scored on a 5-point scale with anchors: 1 (not at all likely), 5 (extremely likely).

Note: *p<.05, **p<.01, ***p<.001.

### Escorted drivers vs. Unescorted walkers

Children are significantly less likely to be driven to school than to walk unescorted if they are older, *B*=−0.88, *OR*= 0.42, Wald χ^2^ (1)=63.55, *p* <.001, if the predominant language spoken at home is English, *B*=−1.45, *OR*= 0.23, Wald χ^2^ (1)=10.49, *p* <.001, and if their current household is within one kilometer from school, *B*=−2.24, *OR*= 0.11, Wald χ^2^ (1)=24.91, *p* <.001. Children are marginally more likely to be driven to school than walk unescorted with each additional household car owned, *B*=0.69, *OR*= 2.00, Wald χ^2^ (1)=3.80, *p* = .05.

#### Group-wise comparisons on parental perceptions/attitudes

##### Escorted walkers vs. Unescorted walkers

Relating to perceptions of AST safety, the more likely parents worry about strangers and bullies approaching their child, the more likely their child walks escorted as opposed to unescorted, *B*=0.26, *OR*= 1.30, Wald χ^2^ (1)= 7.78, *p* <.01. Additionally, the older the child (*M*_age_ = 11.46, Median_age_ = 11.00) that parents would allow their child to walk to school unescorted, the more likely that their child is currently escorted while walking to school as opposed to walking unescorted, *B*= 0.37, *OR*= 1.45, Wald χ^2^ (1)= 18.22, *p* <.001. Although not displayed in either table, our analysis showed that parents of escorted walkers indicate they would allow their child to travel to/from school unescorted at a mean age of 11.5, while the parents of the unescorted walkers allowed their child to walk unescorted at the mean age of 10.08. Results also reveal that the more parents agree that they have discussed AST related safety precautions with their child, the less likely their child was to walk escorted, *B*=−0.38, *OR*= 0.69, Wald χ^2^ (1)=3.91, *p* <.05. There were no statistically significant findings between the children who walk escorted versus unescorted with regard to parental perceptions of AST benefits (Table 2).

### Escorted drivers vs. Unescorted walkers

Relating to perceptions of AST safety, children are more likely to be driven to school than to walk unescorted the more their parents agree that there are too many cars in the morning around school, *B*=0.28, *OR*= 1.31, Wald χ^2^ (1)=5.17, *p* <.001 and the more they worry about strangers/bullies approaching their child, *B*=0.46, *OR*= 1.58, Wald χ^2^ (1)=11.96, *p* <.05. Additionally, the older the age (*M*_age_ = 11.84, Median_age_=12.00) that parents would allow their child to walk to school unescorted, the more likely that their child is currently driven to school as opposed to walking unescorted, *B*=0.39, *OR*= 1.48, Wald χ^2^ (1)=13.26, *p* <.001. Results also reveal that the greater parents agree that they have discussed AST related safety precautions with their child, the less likely their children are driven to school opposed to walking unescorted, *B*=−1.02, *OR*= 0.36, Wald χ^2^ (1)=28.82, *p* <.001. Alternatively, children are 2.78 times more likely to walk to school unescorted than be driven if their parents have discussed AST related safety precautions with their child. Regarding parental perceived AST advantages, children are less likely to be driven to school if their parents value the exercise attained en route to/from school, *B*=−0.95, *OR*= .39, Wald χ^2^ (1)=17.75, *p* <.001, and the more they agree that they chose to live in their current area so that their child can walk to school, *B*=−0.42, *OR*= 0.66, Wald χ^2^ (1)=13.41, *p* <.001.

### Methods of increasing AST among escorted drivers

**Figure 1:**
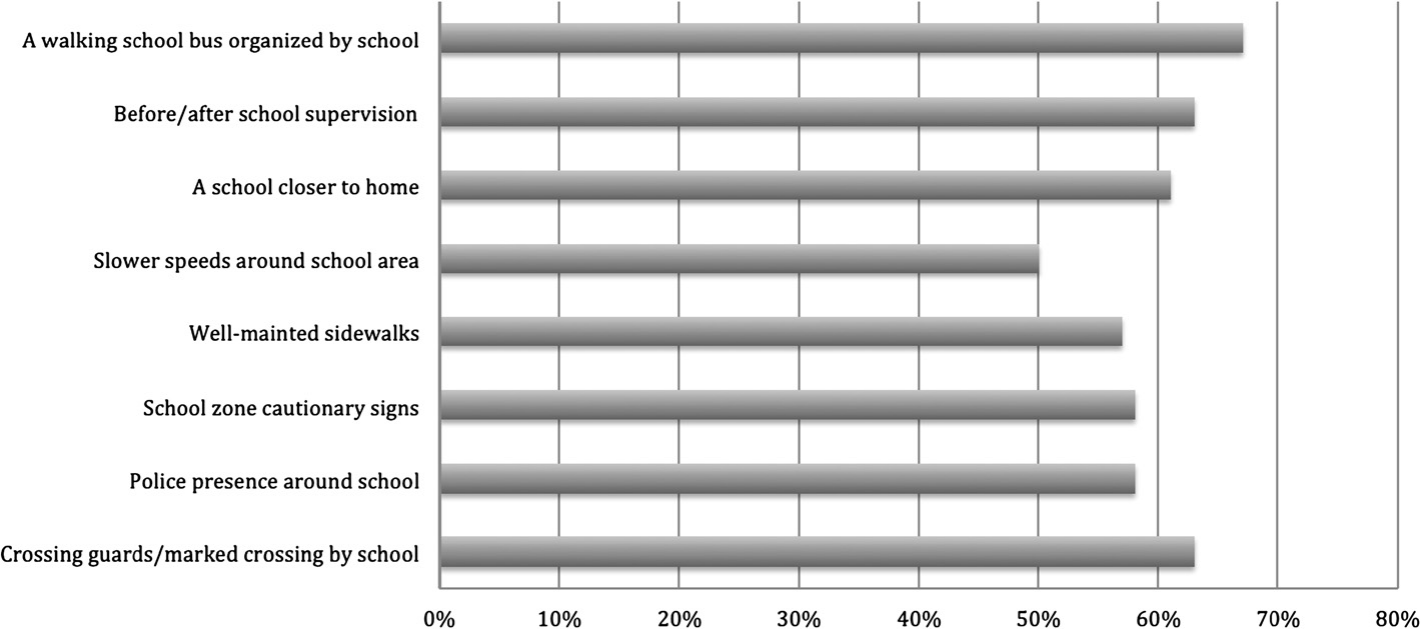
Strategies to increase AST among drivers.

Notably, parents of the escorted drivers were more likely to endorse the appeal of walking to school with their child (*B*=0.37, *OR*= 1.44, Wald χ^2^ (1)=4.03, *p* <.05), and would be more likely to utilize an organized walking school bus (*B*=0.26, *OR*= 1.30, Wald χ^2^ (1)=3.92, *p* <.05). As shown in Figure 1, 67% of parents who drive their children to/from school indicated that using a walking school bus organized by the school would increase the likelihood of their child practicing AST. Additionally, 63% of these parents reported that having crossing guards, marked crossings near the school, and having before and after school supervision (i.e., walking escorted) would also increase the likelihood of their child walking/biking to and from school. Parents also indicated that a school closer to home (61%), police presence around the school (58%), having school zone cautionary signs posted (58%), having well maintained sidewalks (57%), and seeing slower speeds around the school area (50%) would increase the likeliness of their child practicing AST.

## Discussion

One of the primary objectives of this study was to draw comparisons between unescorted (walking) and escorted (walking or driven) children in a sample living within two kilometers from school with regard to compositional and contextual dimensions, and parental AST related perceptual differences. This comparative cross-sectional analysis was designed to produce novel insights into how we may shift school travel mode choice from driving to walking so that children can experience the PA and independent mobility benefits of unescorted AST trips. A second objective was to examine methods and strategies that could increase the likelihood of AST among the escorted drivers.

In terms of demographics, unescorted walkers were significantly older and the families spoke predominantly English at home, and were more likely to live within one kilometer from school. Although previous research has shown mixed findings in terms of the association between age and AST, recent studies have shown age to be significantly associated with children’s independent mobility to/from school [39], and that older children are less likely to be escorted to/from school by their parents [34,35]. Our finding appears to imply that there may be an age threshold where parents believe that their child has the cognitive capacity to navigate his/her way to school safely. Results showed that parents of children who are escorted to school (walked or driven) would only allow their child to walk unescorted at the mean ages 11.5 (currently walking escorted), and 11.84 (currently driven), respectively, when the child is approaching junior high school. Any age lower than the threshold may be viewed by parents as being an unsafe age for children to actively travel to school without adult supervision. This is supported in previous literature, which suggests that younger children are at higher risk when exposed to traffic situations [40] due to their attentional skills and their age-moderated appetite for risk taking [41,42]. Additionally, parents may feel that an older child possesses a greater sense of agency, and is therefore more capable of advocating for her/his own safety when in the presence of strangers.

The GTHA is composed of the metropolitan areas of the cities of Toronto and Hamilton, Ontario along with the regional municipalities of Durham, Halton, Peel and York. This region has varying built environments spread across traditional downtown, inner and outer suburban areas. Notably, region, at the broad scale applied here, did not differentiate those who walked unescorted from those who were escorted. Rather, a child living within one kilometer from school was more likely to walk unescorted regardless of regional location. We expect, however, that marked difference could emerge with a shift in scale that would permit neighborhood by neighborhood comparison within an individual city or regional municipality.

Distance is consistently found to be negatively associated with AST [20]. Our findings highlight that, even in a sample living within a ‘walkable’ distance from school, distance remains a strong predictor of both travel mode and independent travel. Similarly, Fyhri and Hjorthol [39] reported that increasing the distance to school from one to two kilometers was associated with a significant decrease in independent mobility. A reduced distance from school may help parents feel more at ease with their child walking unescorted due to a reduction in the time exposed to risk (e.g., fewer streets to cross, fewer opportunities to run into strangers on a short trip). To promote AST, these findings reinforce the fundamental importance of ensuring school catchment areas are proximate to schools [43,44].

This study also portrayed that the families of children who were unescorted spoke predominantly English at home when compared with families of the children who were escorted to school. Given that there were no differences in terms of household income, this finding may suggest ethnic or cultural differences in independent mobility, in line with previous research showing that minority ethnic children were more restricted in their independence [45]. Future research might be informative in considering cultural variations in perceptions of independent mobility and school travel. Giuliano [46] noted that understandings about travel behavior have historically focused on Caucasians, with less attention given to the question of how race/ethnicity manifests itself within location decisions and travel behavior. More work is needed to understand the travel motivations, constraints, and travel outcomes of various ethnic groups. Certainly, in the presence of an increasingly complex landscape of diversity within cities and regions, some careful attention (in research addressing travel behavior) should be given to how we define difference, and how difference produces or is produced by travel behavior decisions and outcomes. Data limitations prevent such detailed analysis in this work, where instead, the primary language spoken at home is used as a measure of language background. The authors acknowledge that while this variable relates to culture, it is an incomplete indicator of, for example, race and/or ethnicity [47,48].

As a novel contribution, we also found that children were more likely to walk unescorted to/from school if their parents agreed to a greater extent that they chose to reside in the current neighborhood in order for their child to practice AST. Those families inclined to walk for transportation in general, or who are interested in their children walking to school may seek out and eventually reside in neighborhoods that are more conducive (e.g., closer) to practicing AST and achieving independent mobility. This suggests that neighborhood self-selection may be an important correlate to consider when studying AST. That is, higher rates of AST for example might not be caused by built environment characteristics that support AST in specific neighbourhoods [20]. Rather, parents who attach importance to a physically active lifestyle might select homes and neighbourhoods that are generally walkable in nature (note in our sample, drivers placed less importance on their child exercising en route to/from school). Based on recent reviews in the AST literature [49,50], residential self-selection has not been explicitly discussed and future research examining factors influencing AST should control for residential self-selection in their analyses where possible.

In terms of parents’ perceptions and attitudes related to AST, safety concerns clearly differentiated the unescorted and escorted groups. In general, escorting parents had greater concerns about strangers/bullies approaching their child, and parents who drove their children to school were additionally concerned with traffic volume around school. Fear of child abduction or ‘stranger danger’ [16], neighborhood violence [51,52], and traffic volume [53] are all significant barriers to AST and independent mobility in children. Ironically, driving parents also had greater concerns that there were too many cars in the morning around their child’s school. This concern likely results from the fact that even children that are driven end up pedestrians at the school-end, around the site – and, therefore, experience some exposure to risk that is limited to the destination. Similarly, a recent study found that parents who use their car frequently have higher risk perceptions regarding AST [54]. Parental concerns about traffic safety typically are related to perceptions about the number (traffic volume) and speed of vehicles around the school. In an effort to protect their child, many parents drive their children to and from school. Paradoxically, while they may perceive they are reducing the risk of injury for their own child, they are also contributing to the problem and fear of too many vehicles in the school neighborhood [55].

Given the importance of age, distance and safety issues as significant correlates of independent mobility, non-infrastructure programs and policies should be developed that provide adult supervision. An often-discussed intervention is the Walking School Bus (WSB). Proposed by David Engwicht in the early 1990s, a WSB entails children walking escorted by an adult to and from school with other children. Similar to a school bus, these adult supervisors act as ‘drivers’ and collect children along the route to school and actively escort them in a timely, safe manner. Although a WSB provides adult escorts, shifting school travel modes from driving to walking with an adult escort undoubtedly enhances greater independent mobility compared to being escorted to school by car. This was Engwicht’s original intention; the purpose of initiating a WSB was to foster greater independent mobility by providing initial parental guidance. He envisioned that children would graduate to full independence once parents gained confidence in their child’s ability to navigate his/her way to school. More recently, Engwicht [56] reiterated that it is critically important to view WSB schemes as an intermediary step towards a child’s road to full independence. Further, WSB schemes may help establish walking as a normal, daily behavior which promotes walking as a transportation mode of choice for extracurricular activities during a child’s school years and beyond.

Findings from this study support the need to organize WSBs to promote AST, especially for younger students. Parents of driven children were significantly more likely to utilize an organized WSB. A similar measure from this study provided some cause for optimism in that 67% of parent drivers reported that access to a WSB would increase the chances of their child practicing AST while in general parents of children driven to school were also more likely to endorse the appeal of walking to school with their child. However, given the cross-sectional design of this study, causal relationships cannot be assumed between examined variables. For example, it cannot be assumed that providing access to WSB schemes would assist a shift from being chauffeured to school to supervised walking. While the findings indicate that schemes like WSB would appear to be a logical solution in addressing the safety concerns of parents who currently drive their children to school, they are not necessarily common interventions in Canada [57]. There are many challenges in the sustainability of such schemes, such as parent and volunteer compliance, school support, and investment from key stakeholders [58,59]. Recent research also suggests that WSBs may not be cost-effective [60]. However, this study was limited as cost-effectiveness was examined relative to obesity prevention rather than the broader benefits that more active forms of travel may incur. More research is required in understanding the optimal formation and sustainability of these schemes.

Other strategies might be considered that have the goal of alleviating parental concerns regarding the independent mobility of children. For example, an ‘arrival confirmation’ call/text can be made by school administration after attendance is taken to parents of those who actively transported to school. With increasing number of children owning cellular phones, parents should perhaps program the child’s cell device to enable instant communication with a caregiver with a touch of a key (e.g., ‘1’= mom, ‘2’=dad, ‘3’=police), in case the child is in danger or needs help. Alternatively, the role of a crossing-guard can expand to serve two purposes; 1) to help children cross busy streets in a safe manner or 2) to act as ‘eyes on the street’ as they stroll the streets between the school and surrounding neighborhoods. The identification and promotion of school specific ‘safe’ routes to school that minimize traffic exposure and follow routes with high pedestrian use is another example.

In Canada, strategies like these to increase AST and independent mobility are likely to be embedded within broader School Travel Planning (STP) initiatives (see [57]). STP is a multi-disciplinary, multi-sectoral intervention that engages key stakeholders (i.e., public health, police officials, municipal planners & traffic engineers, school boards, parents and school administrators) to document and evaluate school travel issues and develop and implement a travel or ‘action’ plan that creates environments that are more conducive for children to practice AST to/from school [61]. Stemming from the action plan, for example, schools could develop a WSB. Other strategies could be adopted to reflect some of the strategies endorsed in the current study by parents. For example, 63% of parents of children driven to school indicated that having crossing guards and marked crossings by the school would increase the likelihood of their child walking to/from school. Parents also reported that having police presence around the school, having school zone cautionary signs posted, having well maintained sidewalks, and seeing slower speeds around the school area would increase the likeliness of their child practicing AST. It is not clear whether such features were absent or already exist at the schools of the surveyed parents.

School travel plans could also call for educational activities to highlight the contribution AST makes to overall daily energy expenditure levels of children, and to promote walking and cycling safety. Our findings suggest that escorting parents are not talking to their children about safety issues nor are they endorsing the trip to school as an opportunity for physical activity. Addressing these issues through parental educational programs may be required, particularly since past educational interventions have helped increase rates of AST [62,63]. Although AST typically is associated with walking and biking to school, this study explored group differences in terms of walking since only 1% of the sample cycled to school. Future research would be interesting in examining households where children cycle independently to school.

## Conclusions

From both a policy and research perspective, we have highlighted the value of distinguishing between mode (i.e., walking or driving) and travel independence. In terms of research examining school travel, studies typically associate correlates with the school travel mode choice only [33]. Although the number of children walking to school independently was in the minority (11% of children living within 2 km from school), exploring group differences in terms of demographics, and AST parental attitudes provided some insight into how we may help to increase independent mobility among those children who are living within walking distance yet are still driven to school. Safety concerns are heightened for parents who escort their children but whether these can be attenuated before their child ‘is older’ is not clear. For policy, our findings highlight the need for planning decisions about the siting of elementary schools to include considerations of the impact of catchment size on how children get to and from school. WSB schemes might provide one transitional context for enabling parents who drive their children to allow their child some independent mobility on the school trip. Notably, parents of children who walked to school independently were more likely to have chosen to live in a location where their child could walk to school. This emphasizes that residential self-selection may be an important, but overlooked, consideration within research on AST.

## Abbreviations

PA: Physical activity; AST: Active school transportation; GTHA: Greater Toronto and Hamilton Area.

## Competing interests

The authors declare that we have no competing interests.

## Authors’ contributions

GM and GF analyzed and interpreted the data and developed the first draft of the manuscript. GF and RB contributed to the conception and the design of the study. JL contributed to the survey development and interpretation of the data. All authors provided critical feedback during manuscript development. Each author has read and approved the final manuscript.

## Authors’ information

GM is a doctoral student in the Faculty of Kinesiology and Physical Education, University of Toronto. GF is a Professor in the Faculty of Kinesiology and Physical Education, University of Toronto. RB is an Associate Professor in the Department of Geography, University of Toronto. JL is the Program Coordinator of School-Based TDM at Metrolinx, leading the Stepping It Up school travel planning project (http://www.metrolinx.com/schooltravel).

## Pre-publication history

The pre-publication history for this paper can be accessed here:

http://www.biomedcentral.com/1471-2458/12/862/prepub
